# Systematic discovery of novel ribozymes with gene silencing properties from global deep-sea RNA viromes

**DOI:** 10.3389/fmicb.2026.1866359

**Published:** 2026-08-04

**Authors:** Yuan Zhang, Xiaobo Zhang

**Affiliations:** College of Life Sciences, Laboratory for Marine Biology and Biotechnology of Pilot National Laboratory for Marine Science and Technology (Qingdao), Southern Marine Science and Engineering Guangdong Laboratory (Zhuhai), Zhejiang University, Hangzhou, China

**Keywords:** antiviral activity, deep sea, marine resources, ribozyme, RNA virome

## Abstract

Deep-sea ecosystems harbor extraordinary viral diversity, yet their potential as reservoirs of catalytic RNAs remains unexplored. Here, we systematically analyzed RNA viromes from 291 globally distributed deep-sea sediment samples, spanning seven distinct ecosystems across three oceans, and identified 1,305 distinct hammerhead ribozymes (485 type I, 561 type II, and 259 type III). Functional characterization revealed that ribozyme DP163 cleaved the HIV-1 *pol* mRNA, reducing viral copies and infection. Similarly, ribozyme DP162 suppressed HSV-1 infection by targeting the viral *ICP4* gene, achieving ICP4 knockdown and enhancing antiviral immunity. Engineered variants of DP162 and DP163 demonstrated gene silencing efficiency comparable to siRNA. Our findings establish deep-sea RNA viruses as an untapped reservoir of natural ribozymes with dual functionality: intrinsic antiviral properties through immune activation and programmable gene silencing capacity, providing novel catalytic RNA scaffolds for therapeutic development and synthetic biology applications.

## 1 Introduction

The deep-sea environment, encompassing over 60% of Earth's surface area and representing the planet's largest continuous habitat, remains among the most poorly characterized ecosystems in terms of its microbial and viral diversity (Neri et al., 2022). Despite extreme physicochemical conditions including elevated hydrostatic pressure (>100 atm), low temperatures (2–4 C), and complete absence of sunlight, deep-sea ecosystems harbor extraordinary microbial diversity, with viral particles often exceeding 107 per milliliter of seawater (Zhang et al., 2024). Deep-sea viruses are critical drivers of biogeochemical processes and ecological dynamics in deep ocean ecosystems (He et al., 2024; Zhang et al., 2024). These observations raise a fundamental question: do deep-sea RNA viruses harbor novel catalytic RNA elements adapted to extreme conditions? Recent advances in viral metagenomics have revealed that deep-sea environments harbor extraordinary viral diversity, with RNA viruses representing a significant yet understudied component of the deep-sea virome (Zhang et al., 2024). These RNA viral communities exhibit distinct taxonomic compositions compared to their surface ocean counterparts, suggesting unique evolutionary pressures and ecological adaptations in deep-sea environments (Zhang et al., 2024).

The discovery of catalytic RNAs in extreme environments holds particular significance in the context of the RNA World hypothesis, which posits that self-replicating ribozymes preceded modern protein-based life (Higgs and Lehman, 2015). Extreme environments such as deep-sea hydrothermal vents may resemble early Earth conditions, potentially preserving ancient or biochemically distinct catalytic RNA lineages (Fine and Pearlman, 2023). Ribozymes, small catalytic RNAs typically 30–150 nucleotides in length, catalyze sequence-specific phosphodiester bond cleavage, often with divalent metal ion assistance (Weinberg et al., 2019). Hammerhead ribozymes (HHRs) were first identified in the satellite RNA of tobacco ringspot virus and the avocado sunblotch viroid (Hutchins et al., 1986; Prody et al., 1986), and are now recognized as one of several classes of natural ribozymes (Park et al., 2019; Weinberg et al., 2019). At present, the known natural ribozymes contain hammerhead, hairpin, hepatitis delta virus (HDV), and self-splicing introns, distributed across eukaryotes, prokaryotes, bacteriophages, and viruses (Park et al., 2019; Weinberg et al., 2019). The catalytic efficiency of HHRs depends not only on the conserved catalytic core but also on peripheral structural elements and long-range tertiary interactions between the loops capping stems I and II (McDowell et al., 2010; O'Rourke et al., 2015). These loop–loop contacts stabilize the catalytically active conformation and enable efficient cleavage at physiologically relevant Mg^2+^ concentrations, properties that are absent in minimal HHR constructs and are critical for intracellular activity (McDowell et al., 2010; O'Rourke et al., 2015). The extended hammerhead ribozymes have been identified in the genomic contexts of diverse organisms, including viral genomes and eukaryotic untranslated regions, where they are proposed to participate in RNA processing and post-transcriptional gene regulation (Martick et al., 2008). Notably, hammerhead ribozymes have been identified in viral genomes, including the5′-untranslated regions of RNA viruses and adjacent to tRNAs in bacteriophages (Kienbeck et al., 2024; Liu et al., 2024), suggesting roles in viral replication and host interaction. Over the past decades, numerous ribozymes have been discovered. The sequence-specific RNA cleavage activity of HHRs makes them attractive candidates for therapeutic gene silencing, as they can be engineered to target and cleave disease-relevant transcripts in a catalytic, protein-independent manner (O'Rourke et al., 2015; Park et al., 2019). Despite their therapeutic potential, comprehensive surveys of ribozyme diversity in environmental viral communities remain limited. Most characterized ribozymes originate from mesophilic organisms or laboratory selections, leaving extreme environment-derived variants largely unexplored (Weinberg et al., 2021). This knowledge gap is particularly significant given that extremophile-derived ribozymes may exhibit enhanced stability, unique substrate specificities, or novel catalytic mechanisms adapted to harsh conditions. Moreover, natural ribozymes serve as essential scaffolds for engineering gene silencing tools: optimized hammerhead ribozymes achieve catalytic mRNA cleavage, potentially offering advantages over small interfering RNA (siRNA)-based approaches, which require the assembly of RNA-induced silencing complex (RISC), a multiprotein machinery that degrades target mRNAs in a protein-dependent manner (Kobori et al., 2015; Liu et al., 2024). The recent development of hydrolytic endonucleolytic ribozyme (HYER), engineered catalytic RNAs capable of sequence-specific DNA cleavage without protein cofactors, for protein-free DNA editing further underscores the biotechnological value of discovering natural ribozyme variants (Liu et al., 2024). Trans-cleaving hammerhead ribozymes have been employed as gene-silencing tools in mammalian cells by targeting disease-relevant transcripts through sequence-specific RNA cleavage at NUH trinucleotide motifs, where N is any nucleotide and H is A, C, or U, the minimal cleavage recognition sequences of hammerhead ribozymes (Liu et al., 2017). The engineered trans-acting ribozymes have demonstrated effective knockdown of both exogenous and endogenous RNAs across multiple mammalian cell models (Huang et al., 2019). Further advances include their application in controlling T cell proliferation and their integration with CRISPR-based RNA-targeting systems (Chen et al., 2010; Zhan et al., 2023), highlighting the broad therapeutic potential of ribozymes in mammalian systems.

To characterize natural ribozymes from RNA viruses, a systematic survey of ribozymes from deep-sea RNA viral communities was performed in this study based on the deep-sea RNA viromes obtained from 291 deep-sea sediments belonging to 7 distinct deep-sea ecosystems (seamounts, ocean basins, cold seeps, hydrothermal vents, hadal trenches, polymetallic nodule areas, and mid-ocean ridges) spanning the Pacific, Atlantic and Indian Oceans. The results showed that the natural ribozymes DP162 and DP163 presented gene silencing properties. DP163 possessed the potential for loop-loop interactions between terminal loops, a hallmark of extended-type natural HHRs, while DP162 lacked obvious sequence signatures of inter-loop complementarity, conforming a minimal HHR architecture. Our findings revealed that deep-sea RNA viruses constituted a rich, previously untapped reservoir of catalytic RNAs with both fundamental biological significance and practical biotechnological applications.

## 2 Results

### 2.1 Global survey identifies novel hammerhead ribozymes in deep-sea RNA viromes

**Figure 1:**
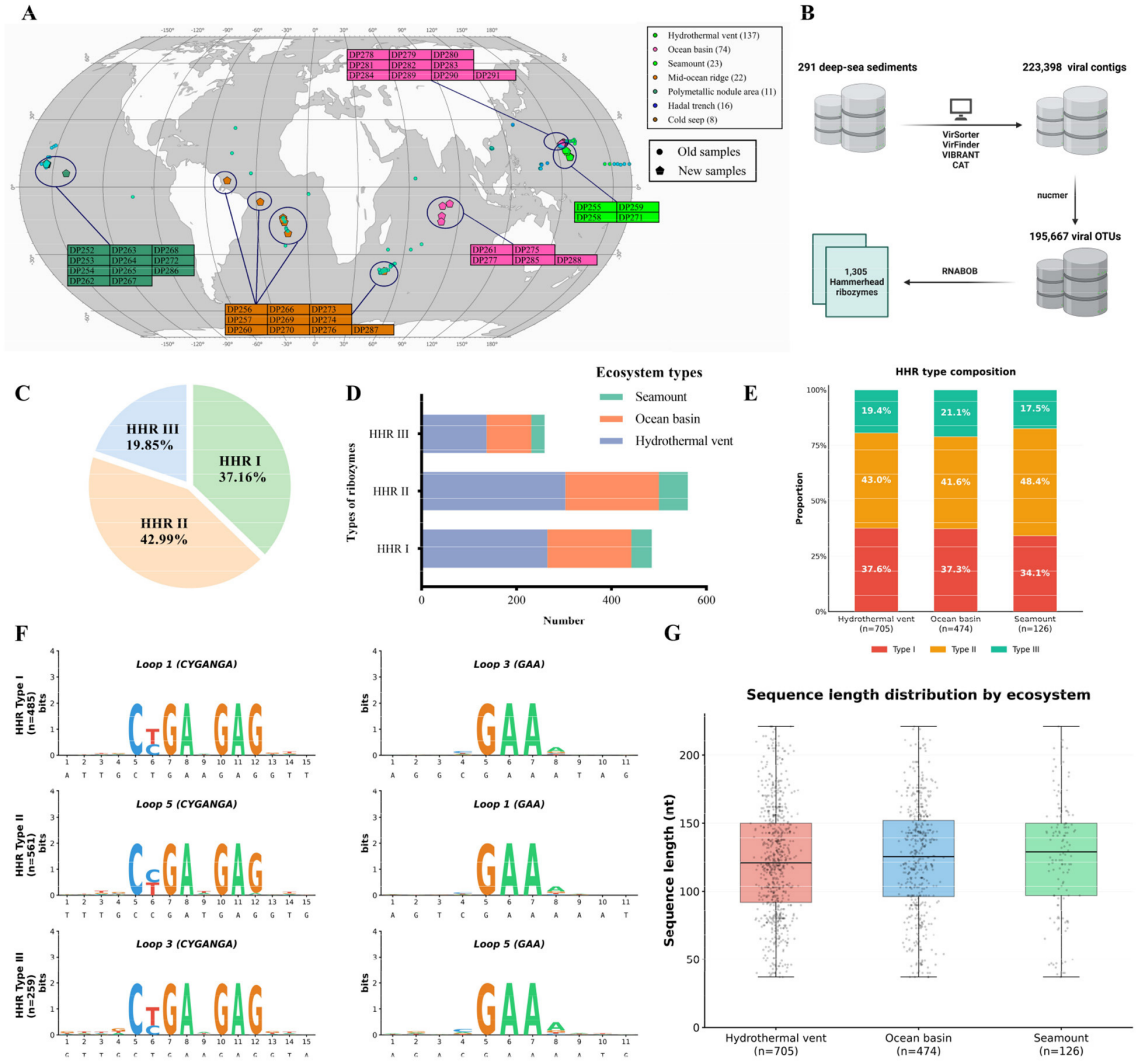
Discovery and characterization of ribozymes in the deep sea. **(A)** Geographic distribution of the sampling stations. A total of 291 sediment samples were collected from 7 deep-sea ecosystems across 3 oceans. Different colors indicated distinct deep-sea ecosystems. The number of samples was shown in the parenthesis. **(B)** Hammerhead ribozymes identified from deep-sea RNA viruses. Based on virome dataset of the RNA viruses purified from each of 291 deep-sea sediments, a total of 1,305 hammerhead ribozymes were obtained. **(C)** Number of hammerhead ribozyme type I (HHR I), HHR II, and HHR III. **(D)** Distribution of hammerhead ribozymes in different types of ecosystems. **(E)** Composition of HHR types across three deep-sea ecosystems. The stacked bar chart illustrated the proportional distribution of type I, type II, and type III HHRs across different deep-sea ecosystems. **(F)** Sequence logo analysis of conserved catalytic core motifs across three HHR types. Sequence logos illustrating nucleotide conservation at the CYGANGA and GAA catalytic core motifs in three HHR types were shown with ±4 flanking nucleotides. The CYGANGA motif was located in loop 1, 5, and 3 for type I, II, and III, respectively. The GAA motif was located in the corresponding loop 3, 1, and 5. The y-axis represented information content (bits), with taller letters indicated higher positional conservation. Nucleotides were colored as follows: A (green), C (blue), G (orange), and T (red). **(G)** Sequence length distribution of HHRs across three deep-sea ecosystems. Box plots showed the sequence length distributions of HHRs in the hydrothermal vent, ocean basin, and seamount. The box represented the interquartile range (IQR, Q1–Q3) and the horizontal line indicated the median.

To explore ribozymes in the deep ocean, the virome datasets of RNA viruses purified from each of 291 deep-sea sediment samples were characterized. These sediment samples were globally distributed across 7 ecosystems (seamount, ocean basin, cold seep, hydrothermal vent, hadal trench, polymetallic nodule area, and mid-ocean ridge) of the Pacific, Atlantic and Indian Oceans (Figure 1A; Supplementary Figure S4). Among the 291 sediment samples, the RNA viromes of 251 sediments had been analyzed in our laboratory previously (Zhang et al., 2024b,a) [National Omics Data Encyclopedia database (accession numbers OEP002537 and OEP003296)] and the RNA virome of the remaining 40 sediments was obtained in the present study (National Omics Data Encyclopedia database accession number OEP00006529).

Based on the RNA virome datasets of 291 deep-sea sediments, a total of 195,667 viral operational taxonomic units (vOTUs) were identified (Figure 1B), representing the most comprehensive deep-sea RNA virome dataset to date. Using RNABOB with hammerhead ribozyme-specific descriptors (see Methods), we identified 1,305 putative ribozyme sequences, comprising 485 hammerhead ribozyme type I (HHR I), 561 HHR II and 259 HHR III (Figure 1C, Supplementary Tables S1–S3).

Ecological distribution analysis revealed that ribozymes were predominantly found in three ecosystem types: hydrothermal vents (705 sequences: 265 type I, 303 type II, and 137 type III), ocean basins (474 sequences: 177 type I, 197 type II, and 100 type III), and seamounts (126 sequences: 43 type I, 61 type II, and 22 type III; Figure 1D).

To examine the compositional variation of hammerhead ribozyme (HHR) types across different deep-sea ecosystems, HHR sequences detected from three ecosystems were categorized by type and their relative proportions. The results indicated that type II HHR was the most abundant across all ecosystems (hydrothermal vent, 43.0%; ocean basin, 41.6%; seamount, 48.4%), followed by type I (hydrothermal vent, 37.6%; ocean basin, 37.3%; seamount, 34.1%) and type III (hydrothermal vent, 19.4%; ocean basin, 21.1%; seamount, 17.5%; Figure 1E). Chi-square analysis revealed no significant difference in HHR type distribution among the three ecosystems (*p* = 0.69), showing that the relative abundance of HHR types was highly conserved across deep-sea ecosystems without ecosystem-specific enrichment patterns.

To investigate the sequence conservation of HHR catalytic core, multiple sequence alignment was performed on a total of 1,305 HHR sequences, and the nucleotide composition and conservation at each position were visualized using sequence logos. The results demonstrated that all three HHR types exhibited near-absolute conservation at the core positions of both the CYGANGA and GAA regions, where each position was occupied by a single dominant nucleotide, while flanking regions showed minimal conservation (Figure 1F). Within the CYGANGA motif, the five core nucleotides C, G, A, G, and A were highly consistent across all three types, with only position 6 (the Y position) showing C/T degeneracy (Figure 1F). The GAA motif likewise displayed strict conservation of G, A, and A across all types (Figure 1F).

To explore potential differences in HHR sequence length across deep-sea ecosystems, statistical analyses were conducted on sequences from three ecosystems, including hydrothermal vent (*n* = 705), ocean basin (*n* = 474), and seamount (*n* = 126). The mean sequence lengths were 122.1, 123.7, and 125.0 nt, with medians of 121, 126, and 129 nt, for hydrothermal vent, ocean basin and seamount, respectively (Figure 1G), indicating highly consistent sequence length distributions across ecosystems.

Taken together, these findings presented that 1,305 novel ribozymes are identified from deep-sea RNA viromes, showing that deep-sea RNA viruses serve as a reservoir of ribozymes.

### 2.2 Ribozyme DP163 suppresses HIV-1-based VSV-G pseudotyped lentivirus infection through *pol* mRNA cleavage

**Figure 2:**
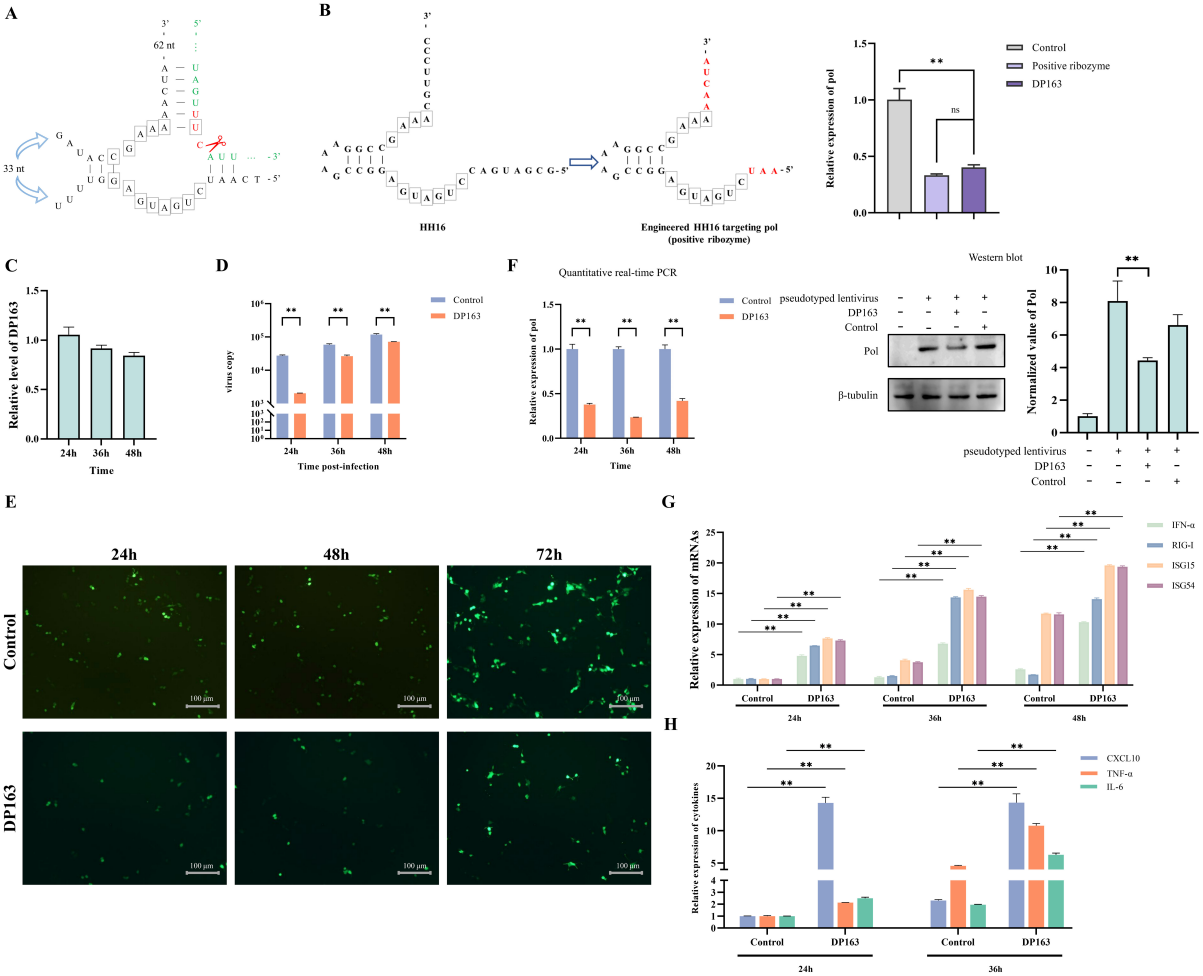
Antiviral activity of deep-sea ribozyme DP163 targeting HIV-1 *pol* gene. **(A)** Secondary structure of the ribozyme DP163. The ribozyme sequence was shown in black and the target *pol* mRNA of HIV-1 was indicated in green. The cleavage site was indicated by a red scissor. Essential catalytic residues were highlighted with boxes. **(B)** Confirmation of the catalytic activity of ribozyme DP163. The substrate-binding arms of a known ribozyme HH16 were engineered to target the *pol* gene. The changed nucleotides were indicated with red. The engineered HH16 was used as a positive control ribozyme (positive ribozyme). HEK-293T cells were transfected with DP163 or the positive ribozyme. Six hours later, the cells were co-transfected with the lentiviral packaging plasmids psPAX2 and pMD2.G together with PLVX-shRNA to initiate lentiviral packaging. At 24 h after transfection, the expression level of the *pol* gene was determined (**, *p* < 0.01). Random RNA was used as a control. **(C)** Existence of ribozyme DP163 in HEK-293T cells. HEK-293T cells were transfected with the ribozyme DP163. Cells treated with random RNA served as a control. At different time after transfection, the intracellular DP163 level was quantified using quantitative real-time PCR, with the 24 h time point serving as the reference for normalization of DP163 level (**, *p* < 0.01). **(D)** Influence of ribozyme DP163 on virus infection. HEK-293T cells were transfected with DP163 and cultured for 6 h, followed by the co-transfection of PLVX-shRNA, psPAX2, and pMD2.G to generate lentiviruses. Cells transfected with random RNA was used as a control. At different time after transfection, the virus copies were quantified using quantitative real-time PCR (**, *p* < 0.01). **(E)** Fluorescence-based detection of HIV-1-based VSV-G pseudotyped lentivirus infection using fluorescence. HEK-293T cells were transfected with ribozyme DP163 and cultured for 6 h, followed by co-transfection of lentiviral packaging plasmids. As a control, a random RNA was included in the assays. Forty-eight hours later, the supernatant was collected, filtered, and concentrated to harvest viral particles. The packaged lentivirus carried ZsGreen, a green fluorescent protein, encoded by PLVX-shRNA. The green fluorescence in newly infected cells directly reflected the quantity of functional lentivirus produced. Fluorescence was examined at 24 h, 48 h, and 72 h post-infection. Scale bar, 100 μm. **(F)** Cleavage of *pol* mRNA by ribozyme DP163 in cells. HEK-293T cells were transfected with DP163 or random RNA (control), followed by the co-transfection of PLVX-shRNA, psPAX2, and pMD2.G. At different time after transfection, the *pol* mRNA level was examined using quantitative real-time PCR (**, *p* < 0.01). Pol protein was detected using Western blot at 48 h after transfection. β-tubulin served as a control. The protein band intensities were normalized to β-tubulin by densitometric analysis using Image J (https://ij.imjoy.io/) to quantify pol protein (**, *p* < 0.01). **(G)** Effects of ribozyme DP163 on the interferon pathway. HEK-293T cells were transfected with ribozyme DP163. At different time after transfection, the expression levels of RIG-I, interferon-stimulated genes (ISGs) and interferons (IFNs) were detected using quantitative real-time PCR (**, *p* < 0.01). Cells treated with random RNA were used as a control. **(H)** Influence of DP163 on cytokine expressions in cells. The DP-163-treated HEK-293T cells were subjected to quantitative real-time PCR to detect the expressions of cytokines at different time after treatments (**, *p* < 0.01). Cells treated with random RNA were used as controls.

To explore the antiviral activity of deep-sea ribozymes, HIV-1 and HSV-1 were selected as target viruses based on their global clinical significance and the well-established potential of ribozymes as antiviral gene-silencing tools. The flanking arms of all 1,305 HHR sequences were examined for Watson-Crick complementarity to HIV-1 and HSV-1 mRNA sequences. Candidate ribozymes were evaluated based on two primary criteria: (1) length of predicted complementarity between flanking arms and target mRNA; (2) position of the cleavage site within a functionally critical region of the viral transcript. DP163 and DP162 were selected as representative candidates that best satisfied both criteria, with cleavage sites positioned within the *pol* gene (encoding the viral polyprotein responsible for HIV-1 replication) and the *ICP4* gene (an essential immediate-early transcriptional regulator of HSV-1), and additionally represent distinct deep-sea ecosystems (hydrothermal vent and ocean basin, respectively), broadening the scope of functional characterization. The results showed that DP163, originally identified from a hydrothermal vent sediment sample, exhibited the longest continuous base-pairing to the HIV-1 pol transcript. The predicted DP163-pol interaction showed complementary base pairing in both flanking stems with the cleavage site positioned at a specific nucleotide of polnnnnnn (Figure 2A).

To confirm the activity of ribozyme DP163, a known ribozyme HH16 was engineered by changing its substrate strand to target HIV-1 *pol* gene, encoding the viral polyprotein responsible for reverse transcription and integration (Figure 2B), which was used as a positive ribozyme as described before (Hertel et al., 1994). DP163 or a positive control ribozyme (positive ribozyme) was transfected into HEK-293T cells. At 6h after transfection, the lentiviral packaging plasmids psPAX2, PLVX-shRNA, and pMD2.G were co-transfected into the cells, whereby psPAX2 encoding the *pol* mRNA, and the three plasmids drove the assembly of an HIV-1-based VSV-G pseudotyped lentivirus. Twenty-four hours later, the expression of *po*l mRNA was examined. The results showed that both DP163 and positive ribozyme suppressed the pol expression (Figure 2B), indicating that DP163 possessed enzymatic activity comparable to that of the positive ribozyme.

To assess the influence of ribozyme DP163 on HIV-1-based VSV-G (vesicular stomatitis virus G protein) pseudotyped lentivirus package, DP163 was transfected into lentivirus-infected HEK-293T cells, followed by analysis of virus copies and fluorescence. The results demonstrated that ribozyme DP163 was successfully transfected and detected in cells (Figure 2C). Treatment with ribozyme DP163 significantly decreased virus copies compared to random RNA control (Figure 2D). The lentivirus carried ZsGreen, a green fluorescent protein encoded by the transfer plasmid PLVX-shRNA. The green fluorescence in newly infected cells directly reflected the quantity of lentivirus produced. The green fluorescence intensity in cells infected with lentivirus packaged from DP163-treated cells was dramatically decreased compared to that of the random RNA control at 24, 48, and 72 h post-infection (Figure 2E), further confirming that DP163 effectively inhibited lentiviral packaging. These data indicated that the ribozyme DP163 suppressed HIV-1-based VSV-G pseudotyped lentivirus infection.

To reveal the mechanism by which ribozyme DP163 suppressed HIV-1-based VSV-G pseudotyped lentivirus package and infection, the mRNA level of pol in DP163-treated cells was examined. The results revealed that the ribozyme DP163 significantly decreased the pol expression in cells compared with the control (Figure 2F). To confirm direct catalytic cleavage, we performed *in vitro* assays using purified DP163 according to the standard methods (Hertel et al., 1994; Liu et al., 2017). The results showed that DP163 could cleave the *pol* mRNA (Supplementary Figure S1). These data indicated that DP163 could cleave *pol* mRNA to suppress HIV-1-based VSV-G pseudotyped lentivirus infection.

To explore the influence of the ribozyme DP163 on innate immune responses, HEK-293T cells were transfected with DP163 and then the expressions of retinoic acid-inducible gene I (RIG-I), interferon-stimulated genes (ISGs) and interferons (IFNs, a family of signaling proteins that mediate antiviral immune responses) were examined. Unlike random RNA controls, ribozyme DP163 cleaved viral *pol* mRNA through its catalytic activity. The ribozyme itself and the ribozyme-mRNA could form double-stranded RNAs, which might activate RIG-I to trigger innate immunity. Furthermore, the cleavage products generated bear2′3′-cyclic phosphate termini, a non-canonical RNA end absent in normal cellular transcripts, serving as an immunostimulatory signal to further amplify the antiviral response (Jung et al., 2020). Upon virus infection, cytoplasmic pattern recognition receptor RIG-I detects non-self RNA species to initiate innate immune signaling pathway, leading to the production of type I interferons (IFN-α and IFN-β) through IRF3 activation and pro-inflammatory cytokines (TNF-α, IL-6 and CXCL10) through NF-κB activation. The secreted IFN-α and IFN-β act in an autocrine and paracrine manner to activate the JAK-STAT signaling pathway, driving the transcriptional upregulation of ISGs, including ISG15 and ISG54, which collectively establish an antiviral state in infected and neighboring cells. The results showed that IFN-α and IFN-β were significantly increased in cells treated with DP163 compared with the control (Figure 2G). This induction was accompanied by upregulation of the pattern recognition receptor RIG-I and downstream interferon-stimulated genes ISG15 and ISG54 (Figure 2G). These data demonstrated that the ribozyme DP163 could activate the IFN pathway.

As reported, HIV-1 is capable of maintaining chronic infection in humans by promoting the production of pro-inflammatory cytokines including C-X-C motif chemokine ligand 10 (CXCL10), interleukin-6 (IL-6), and tumor necrosis factor-alpha (TNF-α) to disrupt innate immune responses (Spina et al., 2013). The results showed that the ribozyme DP163 promoted the expressions of CXCL10, TNF-α and IL-6 (Figure 2H). These data indicated that the ribozyme DP163 could enhance immune responses.

Collectively, these findings demonstrated that the ribozyme DP163 could suppress HIV-1-based VSV-G pseudotyped lentivirus package and infection by cleaving viral *pol* mRNA and triggering immune responses.

### 2.3 Ribozyme DP162 inhibits HSV-1 infection via *ICP4* mRNA cleavage and interferon pathway activation

**Figure 3:**
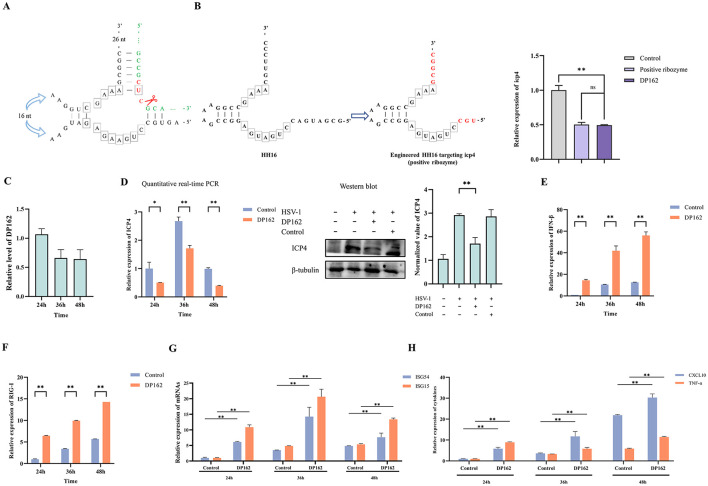
Role of deep-sea ribozyme DP162 targeting HSV-1 transcriptional regulator *ICP4* gene in DNA virus infection. **(A)** Secondary structure of ribozyme DP162. The ribozyme sequence was indicated in black, while the target substrate, the HSV-1 infected-cell polypeptide 4 (ICP4) gene, was shown in green. The cleavage site was marked with a red scissor symbol. Boxes highlighted key catalytic residues. **(B)** Confirmation of the ribozyme DP162. The substrate strand of a known ribozyme HH16 was engineered to target the *ICP4* gene. The changed nucleotides of HH16 were shown in red. The engineered HH16 was included in the assays as a positive ribozyme. HEK-293T cells were transfected with DP162 or the positive ribozyme. Six hours later, the cells were infected with HSV-1 and then cultured for 24 h. Subsequently, the expression level of the *ICP4* gene was determined (**, *p* < 0.01). Random RNA was used as a control. **(C)** Existence of ribozyme DP162 in HEK-293T cells. HEK-293T cells were transfected with DP162, with untreated cells serving as controls. At different time after transfection, DP162 level was examined using quantitative real-time PCR, with the 24 h time point serving as the reference for normalization of DP162 level (**, *p* < 0.01). **(D)** Influence of ribozyme DP162 on the expression of ICP4 in HEK-293T cells. Cells were transfected with DP162 or random RNA (control). Six hours later, the cells were infected with HSV-1. At 24, 36 and 48 h post-infection, the expression of ICP4 was determined using quantitative real-time PCR (*, *p* < 0.05; **, *p* < 0.01). Western blot showed the ICP4 protein level at 48 h post-infection. β-tubulin was used as a control. The protein and band intensities were normalized to β-tubulin by densitometric analysis using Image J (https://ij.imjoy.io/) to quantify ICP4 (**, *p* < 0.01). **(E)** Effects of ribozyme DP162 on the production of interferon. HEK-293T cells were transfected with DP162 or random RNA, cultured for 6 h and then infected with HSV-1. At different time post-infection, the expression level of IFN-β was quantified using quantitative real-time PCR (**, *p* < 0.01). **(F)** Effects of ribozyme DP162 on RIG-I expression. The DP162-transfected HEK-293T cells were subjected to quantitative real-time PCR to examine the expression level of RIG-I (**, *p* < 0.01). Cells treated with random RNA served as a control. **(G)** Impact of ribozyme DP162 on the expression of interferon-stimulated genes (ISGs). The expression levels of ISG54 and ISG15 in the DP162-treated HEK-293T cells were examined using quantitative real-time PCR (**, *p* < 0.01). **(H)** Influence of DP162 on the expression of cytokines in cells. Quantitative real-time PCR was performed to detect the expression levels of CXCL10 and TNF-α in DP162-treated HEK-293T cells at different time points after treatment. Cells treated with random RNA was used as a control (**, *p* < 0.01).

Building on the same screening approach described above, DP162 was identified as the representative candidate that best satisfied both criteria against HSV-1. DP162, originally identified from an ocean basin sediment sample, exhibited the longest continuous base-pairing to the HSV-1 ICP4 (infected cell polypeptide 4) transcript, which encoded an essential immediate-early transcriptional regulator of HSV-1, making it a functionally critical target. The predicted DP162-ICP4 interaction showed complementary base pairing in both flanking stems with the cleavage site positioned at a specific nucleotide of ICP4 (Figure 3A). To confirm the enzymatic activity of DP162, a known ribozyme HH16 was engineered to target the *ICP4* gene using the standard method (Hertel et al., 1994), in which the substrate strand was engineered (Figure 3B). The engineered HH16 was used as a positive ribozyme. DP162 or the positive ribozyme was transfected into the HSV-1-infected HEK-293T cells, followed by the examination of *ICP4* mRNA. The results revealed that DP162 inhibited the ICP4 expression, which was comparable to that of the positive ribozyme (Figure 3B), confirming that DP162 was a ribozyme.

DP162 was successfully transfected into HEK-293T cells, and intracellular RNA levels were confirmed at 24, 36, and 48 h after transfection (Figure 3C). In HSV-1-infected cells, DP162 treatment reduced *ICP4* mRNA levels post-infection (Figure 3D), with corresponding reduction in ICP4 protein (Figure 3D, Western blot). At the same time, *in vitro* cleavage assays, which was performed using the standard methods (Hertel et al., 1994; Liu et al., 2017), revealed that ribozyme DP162 could catalyze the cleavage of *ICP4* mRNA (Supplementary Figure S2). These data indicated that ribozyme DP162 cleaved its target ICP4 mRNA.

To further explore whether DP162 was involved in the IFN pathway, the expression level of IFN-β was examined in the cells treated with HSV-1 and DP162. Given that the ribozyme DP162 cleaved viral *ICP4* mRNA through its intrinsic catalytic activity, generating2′3′-cyclic phosphate-terminated cleavage products absent in random RNA control, it was hypothesized that DP162 might activate RIG-I-mediated innate immune signaling pathway in a manner analogous to DP163. RIG-I activation was expected to induce IFN-β production *via* IRF3, subsequently driving JAK-STAT-dependent upregulation of ISG15 and ISG54, while NF-κB activation would promote the expression of pro-inflammatory cytokines CXCL10 and TNF-α to further enhance antiviral immunity. The results demonstrated that treatment with DP162 significantly enhanced the IFN-β response compared to the control (Figure 3E), showing that ribozyme DP162 could promote IFN production. Simultaneously, treatment with DP162 significantly promoted the expression of RIG-I (Figure 3F). These data demonstrated that ribozyme DP162 triggered RIG-I to promote IFN production. The IFN production induced by ribozyme DP162 further upregulated interferon-stimulated genes (ISG15 and ISG54; Figure 3G) and increased the expressions of CXCL10 and TNF-α (Figure 3H) to enhance antiviral immunity.

Taken together, these results revealed that ribozyme DP162 inhibited DNA virus HSV-1 infection by targeting viral gene and inducing antiviral immunity.

### 2.4 Engineered deep-sea ribozymes achieve programmable gene silencing in mammalian cells

**Figure 4:**
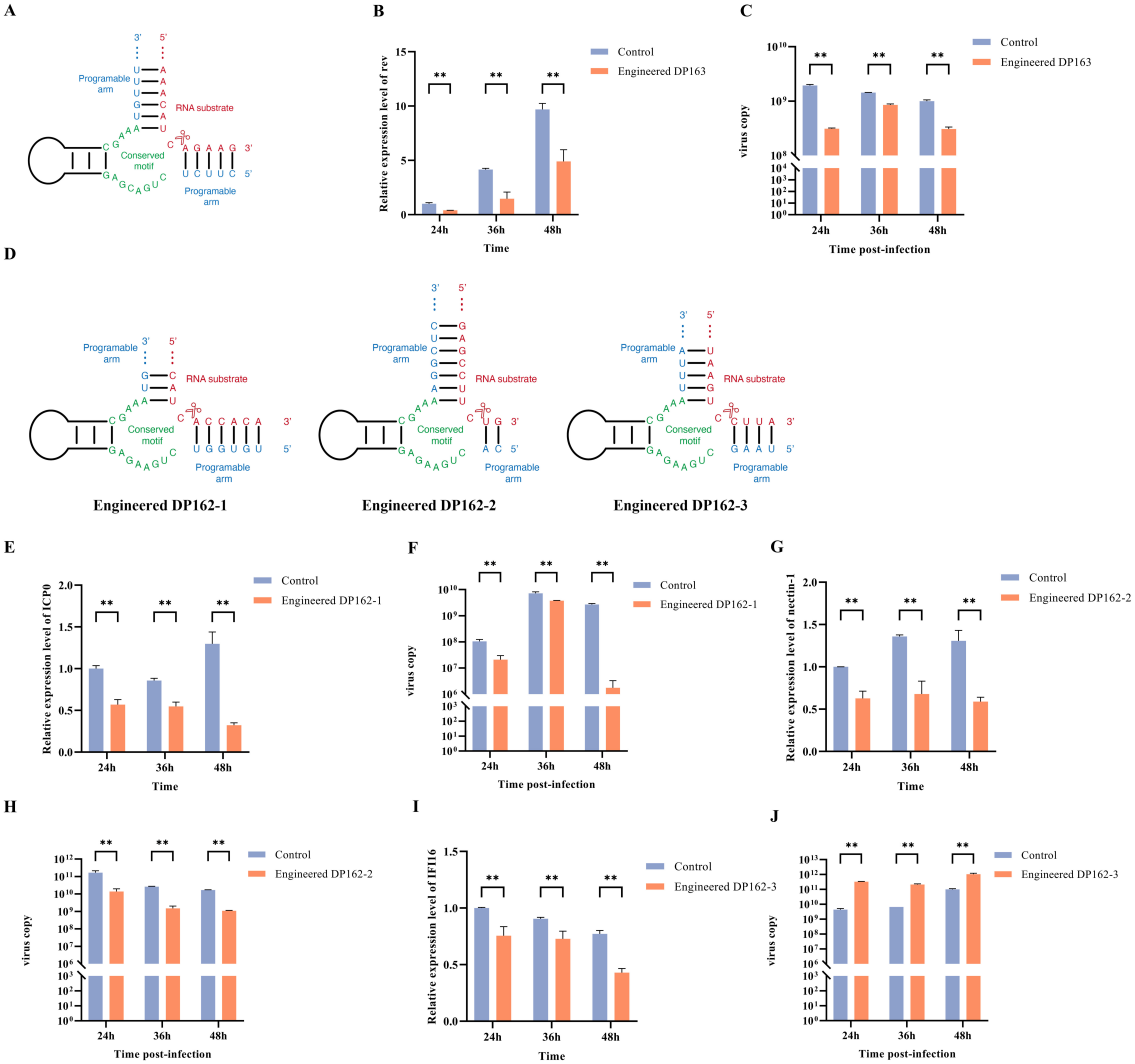
Ribozyme-mediated gene knockdown in cells. **(A)** Schematic representation of engineered ribozyme DP163 targeting the HIV-1 rev gene. The ribozyme contained two programmable arms (blue) that provided sequence-specific recognition of the complementary regions within the RNA substrate (red). The conserved catalytic motif (green) was embedded within the ribozyme structure and was responsible for catalyzing site-specific cleavage of the RNA substrate. **(B)** Knockdown of HIV-1 rev gene by engineered ribozyme DP163. The binding arm sequence of DP163 was engineered to target the HIV-1 rev gene. HEK-293T cells were transfected with engineered DP163. Six hours later, the cells were transfected with the lentiviral packaging plasmids pLVX-shRNA, psPAX2 encoding HIV-1 rev and pMD2.G. Random RNA serv as a control. At different time after transfection, the *rev* mRNA level was quantified using quantitative real-time PCR (**, *p* < 0.01). **(C)** Impact of engineered DP163 on HIV-1-based VSV-G pseudotyped lentivirus package. Virus copy in engineered DP163-treated and virus-infected HEK-293T cells was examined with quantitative real-time PCR (**, *p* < 0.01). Time points post-infection were indicated. **(D)** Schematic diagram of engineered ribozyme DP162. The engineered DP162-1, DP162-2 and DP162-3 targeted ICP0, nectin-1 and ifi16 genes of HSV-1, respectively. Each engineered ribozyme contained two programmable arms (blue) designed for sequence-specific recognition of their respective RNA substrates (red). The conserved catalytic motif (green) was responsible for site-specific cleavage of the target RNAs. **(E)** Silencing of HSV-1 *ICP0* gene by the engineered ribozyme DP162. The engineered DP162-treated HSV-1-infected HEK-293T cells were subjected to quantitative real-time PCR to detect the *ICP0* mRNA (**, *p* < 0.01). **(F)** Quantitative analysis of HSV-1 replication. HEK-293T cells were transfected with the engineered DP162. At 6 h after transfection, the cells were infected with HSV-1. The cells transfected with random RNA were used as controls. The virus copy was determined using quantitative real-time PCR (**, *p* < 0.01). **(G)** Knockdown of the nectin-1 gene mediated by engineered ribozyme DP162 (DP162-2) in cells. HEK-293T cells were transfected with DP162-2 targeting the nectin-1 gene. A random RNA was included in the transfection as a control. At 6 h after transfection, the cells were infected with HSV-1. At different time points post-infection, the nectin-1 mRNA was measured using quantitative real-time PCR (**, *p* < 0.01). **(H)** Quantitative real-time PCR analysis of HSV-1 replication following the engineered ribozyme (DP162-2) treatment. HEK-293T cells were transfected with the engineered DP162-2 for 6 h, followed by the HSV-1 infection. The virus copy in the cells was quantified quantitative real-time PCR at different time points post-infection (**, *p* < 0.01). **(I)** Knockdown of the ifi16 gene using the engineered ribozyme DP162-3 in cells. HEK-293T cells transfected DP162-3 and then infected with HSV-1. At different time post-infection, the expression of ifi16 was examined using quantitative real-time PCR (**, *p* < 0.01). **(J)** Impact of the engineered DP162-3 on HSV-1 replication in cells. HEK-293T cells were transfected with the engineered DP162-3 for 6 h and then infected with HSV-1. The virus copy was determined using quantitative real-time PCR at different time points post-infection (**, *p* < 0.01).

To evaluate whether deep-sea ribozymes could be reprogrammed for arbitrary gene targeting, the ribozyme DP163 was engineered by replacing its flanking arms (stems I and II) to target the HIV-1 rev gene, while preserving the catalytic core (Figure 4A). The engineered DP163 (designated DP163-rev) was designed to cleave rev mRNA (Figure 4A). The results demonstrated that the engineered deep-sea hammerhead ribozyme DP163 significantly downregulated the expression of the HIV-1 rev gene compared to the control (Figure 4B). Consistent with rev knockdown, DP163-rev treatment reduced HIV-1-based VSV-G pseudotyped lentivirus titers (Figure 4C), demonstrating that catalytic activity translates to functional gene silencing.

To validate the generalizability of ribozyme engineering, the ribozyme DP162 was redesigned (DP162-1) to target HSV-1 ICP0 (immediate-early protein 0), a multifunctional E3 ubiquitin ligase that antagonizes host antiviral defenses (Figure 4D). The engineered DP162-1 reduced *ICP0* mRNA of HSV-1 (Figure 4E), resulting in reduction in HSV-1 titers (Figure 4F), confirming that engineered ribozymes achieve gene silencing efficiency.

To further investigate the therapeutic potential of engineered deep-sea ribozymes, DP162 was engineered to target the nectin-1 gene, which encodes the receptor for HSV-1 infection, generating the engineered ribozyme DP162-2 (Figure 4D). The results demonstrated that ribozyme DP162-2 significantly decreased the nectin-1 mRNA level compared to the control (Figure 4G) and significantly reduced HSV-1 copies (Figure 4H). These data demonstrated that the engineered DP162-2 could knock down the expression of its target gene in cells.

To obtain additional data on the gene silencing potential of deep-sea ribozymes, DP162 was further engineered to target the interferon-γ-inducible protein 16 (IFI16) gene, generating the engineered ribozyme DP162-3 (4D). The results showed that DP162-3 suppressed the expression of ifi16 in HEK-293T Fig cells (Figure 4I) and promoted HSV-1 infection in cells (Figure 4J). These data showed that the engineered deep-sea ribozyme was a potential tool to silence gene expression in cells.

Collectively, these findings revealed that deep-sea-derived ribozymes could be engineered to target and cleave the mRNA of specific gene, making them the efficient tools for gene silencing in cells.

### 2.5 Ribozyme-mediated gene silencing requires the catalytic core in mammalian cells

**Figure 5:**
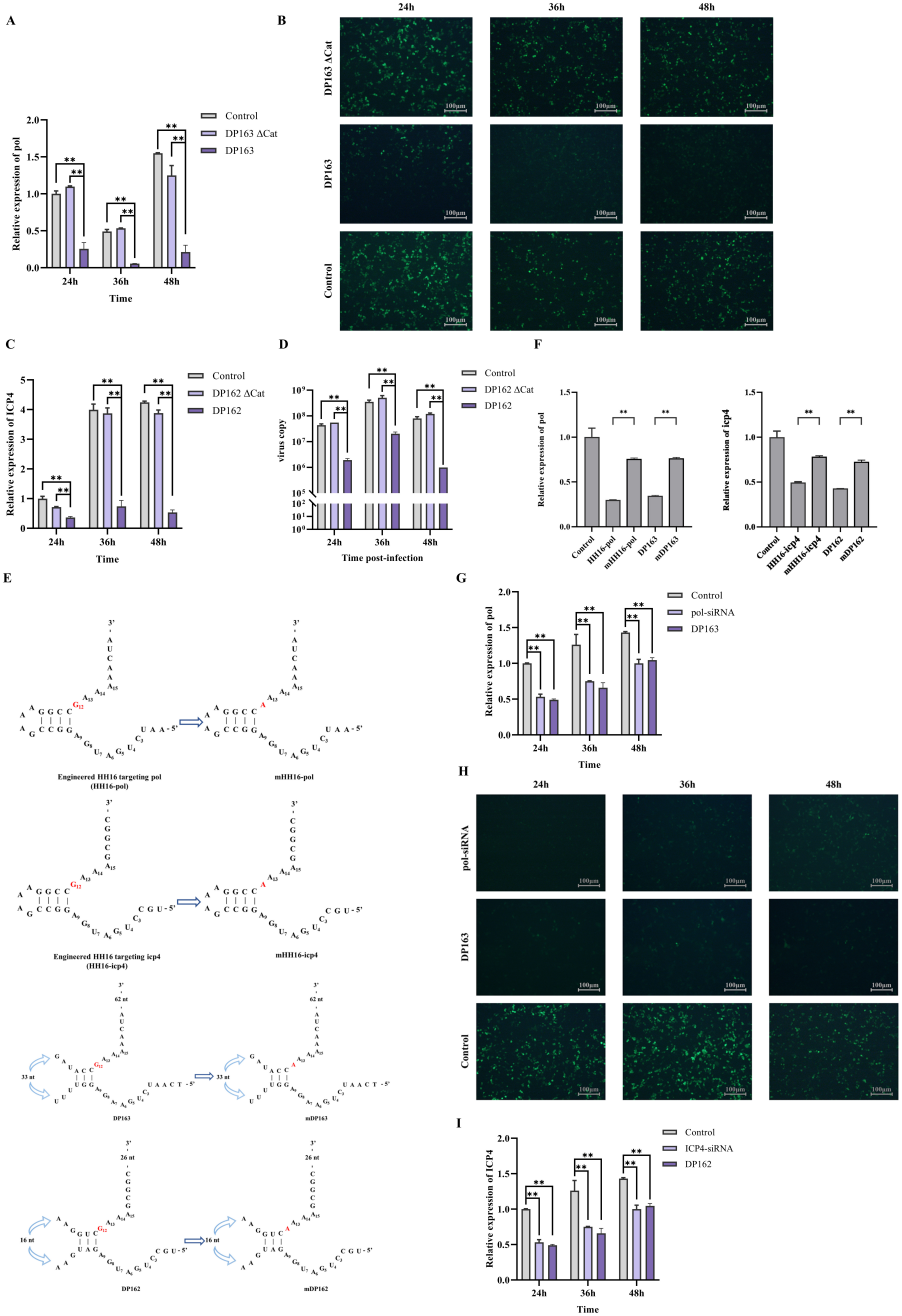
Gene silencing efficiency of deep-sea ribozymes in mammalian cells. **(A)** Role of the catalytic core of ribozyme DP163 in ribozyme activity. HEK-293T cells were transfected with either wild-type ribozyme DP163 or the catalytic core deletion mutant DP163ΔCat. Random RNA served as a control. Six hours later, the lentiviral packaging plasmids psPAX2, pLVX-shRNA and pMD2.G were co-transfected into the cells. At indicated time points post-infection, the *pol* expression was examined using quantitative real-time PCR (**, *p* < 0.01). **(B)** Influence of the catalytic core of ribozyme DP163 on ribozyme activity. HEK-293T cells were transfected with DP163 or DP163ΔCat for 6 h and then transfected with psPAX2, pLVX-shRNA and pMD2.G to drive HIV-1-based VSV-G pseudotyped lentivirus package. The packaged lentivirus was collected and used to transduce target cells. Cellular fluorescence was examined. The numbers indicated the time points post-infection. Scale bar, 100 μm. **(C)** Effects of the catalytic core of ribozyme DP162 on ribozyme activity. HEK-293T cells were transfected with either wild-type ribozyme DP162 or the catalytic core deletion mutant DP162ΔCat, followed by incubation for 6 h. Then the cells were infected by HSV-1. At different time post-infection, the ICP4 expression level was examined using quantitative real-time PCR (**, *p* < 0.01). The cells transfected with random RNA served as controls. **(D)** Antiviral activity of DP162 and DP162ΔCat. HEK-293T cells were transfected with either DP162 or the catalytically inactive mutant DP162ΔCat, followed by an incubation for 6 h prior to HSV-1 infection. Virus copy was quantified by quantitative real-time PCR at different time points post-infection. **(E)** Schematic representation of the active hammerhead ribozymes **(left)** and their corresponding catalytically inactive mutant derivatives **(right)** directed against HIV-1 *pol* mRNA or HSV-1 *ICP4* mRNA. The engineered HH16 (positive ribozyme) targeting pol (HH16-pol), the engineered HH16 targeting ICP4 (HH16-ICP4), DP162 and DP163 were substituted at G12 with A, generating the mutants of ribozymes mHH16-pol, mHH16-ICP4, mDP162, and mDP163. The G12A substitution was indicated in red. **(F)** Catalytic activity of deep-sea ribozymes with G12A substitution. HEK-293T cells were transfected with the indicated ribozymes targeting HIV-1 *pol* mRNA (HH16-pol, mHH16-pol, DP163, and mDP163) or targeting HSV-1 *ICP4* mRNA (HH16-ICP4, mHH16-ICP4, DP162, and mDP162), respectively. Random RNA was included in the assays as a control. At 6 h after transfection, the cells were transfected with the lentiviral packaging plasmids or infected with HSV-1. The cells were cultured for 24 h. Subsequently, the expression levels of pol and ICP4 were examined using quantitative real-time PCR (**, *p* < 0.01). **(G)** Comparison of gene silencing by ribozyme DP163 and siRNA. HEK-293T cells were transfected with DP163 targeting the *pol* gene of HIV-1 or pol-specific siRNA (pol-siRNA), with cells transfected with random RNA serving as controls. At 6 h after transfection, the cells were subjected to lentiviral packaging using the packaging plasmids. At different time after transfection, the expression level of *pol* gene was examined using quantitative real-time PCR (**, *p* < 0.01). **(H)** Comparison of gene silencing by ribozyme DP163 and siRNA assessed by fluorescence microscopy. HEK-293T cells were transfected with DP163 or pol-siRNA. As controls, the cells were transfected with random RNA. At 6 h after transfection, the cells were subjected to lentiviral packaging using the packaging plasmids. The resulting lentivirus was collected and used to transduce HEK-293T cells. At different time after transfection, the fluorescence of cells was examined. Scale bar, 100 μm. **(I)** Gene silencing capacity of ribozyme DP162. HEK-293T cells were infected with HSV-1 and then transfected with DP162 targeting HSV-1 *ICP4* gene or ICP4-specific siRNA (ICP4-siRNA). Random RNA was included in the transfection as a control. At different time after treatment, the expression level of ICP4 in cells was examined using quantitative real-time PCR (**, *p* < 0.01).

To confirm that gene silencing activity depends on catalytic function rather than antisense effects, we generated a catalytically inactive mutant of DP163 (DP163ΔCat) by deleting the conserved catalytic core, while retaining the binding arms complementary to HIV-1 *pol* mRNA. In HEK-293T cells transfected with the lentiviral packaging plasmids psPAX2, pLVX-shRNA and pMD2.G, wild-type DP163 reduced *pol* mRNA, whereas DP163ΔCat showed no significant knockdown (Figure 5A). Correspondingly, DP163 decreased ZsGreen fluorescence, while DP163ΔCat had no effect (Figure 5B). Viral titers confirmed these findings: DP163 reduced HIV-1- based VSV-G pseudotyped lentivirus copies, whereas DP163ΔCat showed no antiviral activity.

To confirm that catalytic core requirement is generalizable across different ribozyme scaffolds, we generated DP162ΔCat by deleting the type I hammerhead catalytic core. In HSV-1-infected cells, wild-type DP162 reduced *ICP4* mRNA, whereas DP162ΔCat showed minimal activity (Figure 5C). Viral titers were suppressed by DP162 but remained unchanged with DP162ΔCat (Figure 5D). These results, consistent with DP163ΔCat data, confirm that catalytic activity is essential for gene silencing across both type I (DP162) and type II (DP163) hammerhead ribozyme scaffolds.

To further verify that the gene-silencing effects of the deep-sea ribozymes were attributed to catalytic cleavage rather than antisense binding, a G12A substitution was introduced into the conserved catalytic core of the HHR motif of the engineered HH16 (positive ribozyme) targeting pol (HH16-pol), the engineered HH16 targeting ICP4 (HH16-ICP4), DP162 or DP163 (Figure 5E). As reported, the G12 residue is critical for coordinating the catalytic metal ion and facilitating phosphodiester bond cleavage; whose substitution with adenosine selectively abolishes catalytic activity while preserving the integrity of the binding arms and the overall RNA architecture (Scott et al., 2013). As shown in Figure 5F, the G12A substitution significantly increased the expression levels of target genes compared with the controls, indicating that the G12A substitution reduced the activity of ribozyme. These data demonstrated that the silencing effects of ribozymes were dependent on catalytic cleavage rather than antisense binding.

To assess the efficiency of gene silencing by ribozymes, the cleavage of target mRNAs mediated by ribozymes and by siRNAs was compared. Currently, siRNA mediated gene silencing is widely used. The results demonstrated that *pol* gene expression was significantly downregulated by pol specific siRNA (pol-siRNA) and by ribozyme DP163 in HEK-293T cells transfected with the lentiviral packaging plasmids, and the efficiency of gene silencing mediated by DP163 was similar to that mediated by pol-siRNA (Figure 5G). At the same time, the fluorescence intensity of the DP163 or pol-siRNA transfected HEK-293T cells transduced with the resulting lentivirus was remarkably decreased compared to the control (Figure 5H). These results demonstrated that the ribozyme DP163 possessed gene silencing efficiency equivalent to that of siRNA.

To further confirm the efficiency of ribozyme mediated gene silencing, HSV-1 infected HEK-293T cells were transfected with the ribozyme DP162 targeting the *ICP4* mRNA of HSV-1 or ICP4 specific siRNA (ICP4-siRNA) and then the expression level of ICP4 was examined. The results showed that ribozyme DP162 exhibited gene silencing efficiency for ICP4 comparable to that of ICP4-siRNA in cells (Figure 5I).

Taken together, these findings revealed that deep-sea derived ribozymes, whose enzymatic activity depended on the catalytic center, possessed the gene silencing capacity comparable to that of siRNAs in mammalian cells.

## 3 Discussion

Hammerhead ribozymes, a specialized class of small self-cleaving RNAs, have emerged as versatile platforms for therapeutic development, precision gene regulation, and synthetic biology through intrinsic sequence specific phosphodiester bond cleavage (Lu et al., 2025). Although the global architecture, tertiary interactions, and peripheral structural elements play important roles in the catalytic efficiency and specificity of ribozymes (O'Rourke et al., 2015), the catalytic activity of hammerhead ribozymes can be governed by the catalytic center, which features a conserved structural motif whose precisely positioned nucleotides enforce the inline geometry required for nucleophilic attack (Scott et al., 2009; Su et al., 2021). The catalytic center of ribozymes utilizes divalent metal ions to stabilize the RNA structure, preorganize the active site, neutralize developing negative charge, and facilitate general acid–base chemistry that to facilitate the nucleophilic attack on the scissile phosphate bond, enabling efficient substrate cleavage (Lee et al., 2009). Based on the catalytic centers and structural frameworks of natural ribozymes, the ribozymes can be engineered to generate artificial variants through the structure guided mutagenesis and *in vitro* selection, yielding expanded substrate scope, improved turnover kinetics, and enhanced intracellular stability (Deng et al., 2023). In this study, the hammerhead ribozymes DP162 and DP163 were engineered to make minimal catalytic constructs containing the catalytic cores. These constructs possessed the functionality of ribozymes, showing that the catalytic cores were the essential components for the activity of hammerhead ribozymes. Our study demonstrated that the engineered ribozymes DP162 and DP163 could be effective ribozymes for gene silencing. As reported, the HYER ribozyme system offers a protein-free alternative to CRISPR-Cas for gene editing (Liu et al., 2024), and the SAM-utilizing ribozyme (SAMURI) enables chemoselective site-directed RNA modification (Chen et al., 2026; Scheitl et al., 2020), exemplifying how natural ribozyme scaffolds can be transformed into versatile biotechnological platforms (Lindley et al., 2024; Liu et al., 2024). Currently, natural ribozymes have become the basis for the engineering of ribozymes (Ebermann and Müller, 2025), making the discovery of natural ribozymes, especially those from unexplored niches, a priority for expanding design scaffolds and evolutionary diversity. In this study, based on the RNA virome datasets of 291 global deep-sea sediments, a total of 1,305 novel ribozymes were identified, which possessed antiviral activity. In this context, our study demonstrates that the RNA viromes from the deep sea could be important reservoirs of natural ribozymes (Lindley et al., 2024). Discovering natural ribozymes from unexplored environmental niches, such as the deep sea, expands the known natural sequence and structural diversity of ribozymes. Each expansion of the natural ribozyme repertoire has historically opened new engineering possibilities that cannot have been anticipated from existing scaffolds alone (Ebermann and Müller, 2025). Thus, the 1,305 hammerhead ribozyme sequences identified in this study from distinct deep-sea ecosystems substantially broaden the catalog of natural ribozyme scaffolds available for biotechnological exploitation. Critically, both DP162 and DP163 are naturally occurring sequences retrieved directly from deep-sea RNA viral metagenomes, rather than laboratory engineered constructs. The observed complementarity between the flanking arms of DP163 and DP162 and the HIV-1 pol and HSV-1 ICP4 transcripts, respectively, does not reflect a co-evolutionary relationship between deep-sea ribozymes and human pathogens. Rather, it represents coincidental sequence matching arising from the inherent sequence plasticity of HHR flanking arms. Unlike the catalytic core, the flanking arms of hammerhead ribozymes are not structurally constrained, and therefore tolerate considerable sequence variation. It is thus statistically plausible that, among 1,305 diverse HHR sequences recovered from global deep-sea sediments, some would exhibit complementarity to any given mRNA sequence by chance. This sequence plasticity, combined with the modular architecture of HHR ribozymes, positions them as potentially programmable antiviral tools that could in principle be engineered to target a wide range of viral transcripts. The sequence of DP163 suggests potential loop–loop interactions between terminal loops, a hallmark of extended type natural HHRs. In contrast, DP162 conforms at the sequence level to a minimal HHR architecture; nevertheless, its deep-sea viral origin distinguishes it from the mesophilic minimal HHRs characterized in prior studies, and noncanonical stabilizing interactions adapted to extreme conditions cannot be excluded.

Due to the capacity of ribozymes to cleave the target mRNAs, the optimized transacting hammerhead ribozymes demonstrate knockdown efficiencies comparable to siRNA-based systems through catalytic (rather than stoichiometric) turnover (Kobori et al., 2015). The gene silencing has achieved significant clinical milestones through RNA interference (RNAi)-based approaches, including inclisiran (Ray et al., 2020), vutrisiran (Adams et al., 2023), and short hairpin RNA (shRNA)-based systems (Fellmann et al., 2013), underscoring the broad therapeutic potential of RNA-based gene silencing. In this study, the results showed that the deep-sea-derived ribozymes could silence the expression of target genes *via* cleaving the target mRNAs in mammalian cells, which was similar to the siRNA-mediated gene silencing, underscoring their potential as protein-independent effectors. It should be noted that the comparison of gene-silencing efficiency between deep-sea ribozymes and siRNAs was limited to a small number of viral targets; comprehensive equivalence benchmarking across diverse genes remains to be performed in the future. Unlike siRNAs, which require the recruitment of proteins for the assembly of the RNA induced silencing complex (RISC), ribozymes function as autonomous catalytic entities capable of sequence specific RNA cleavage *via* intrinsic enzymatic activity without auxiliary protein machinery (Ebenezer et al., 2025). Ribozymes are independent of cellular cofactors, potentially achieving effective gene silencing at substantially lower concentrations compared to the stoichiometric requirements of siRNA approaches, thereby potentially lowering efficacious dosing thresholds (Ebermann and Müller, 2025). Furthermore, ribozymes demonstrate reduced potential for off target effects due to constrained active site geometry and shorter guide requirements (Myers and Sullivan, 2025). The ribozymes, derived from the deep-sea RNA viromes in this study, might govern the virus host interactions *via* gene silencing by selectively attenuating viral replication or modulating host defense transcripts. The results of this study showed that the catalytic core deletion mutants DP162ΔCat and DP163ΔCat, which lacked the conserved catalytic center while retaining the binding arms, failed to reduce the target viral mRNA levels or suppress virus infection, demonstrating that the binding arms alone were insufficient to mediate the observed gene silencing. Furthermore, a single nucleotide substitution at the universally conserved G12 position within the catalytic core of ribozymes (G12A mutant) significantly decreased the ribozyme activity. These results revealed that the antiviral activity of DP162 and DP163 was dependent on ribozyme catalysis rather than antisense hybridization or steric interference. The vast untapped potential of extreme environment RNA viromes for discovering novel biomolecular tools fundamentally challenges our current understanding of the evolutionary constraints and functional diversity of catalytic RNAs in Earth's most remote ecosystems and expands the actionable repertoire for next generation RNA therapeutic and synthetic biology innovation (Lindley et al., 2024). The deep-sea RNA virome thus represents a discovery platform for catalytic RNA diversity, and the value of this resource lies in the breadth of novel natural scaffolds it provides for the engineering of next generation RNA based therapeutic and synthetic biology tools.

## 4 Materials and methods

### 4.1 Deep-sea sediment samples and virome datasets of deep-sea RNA viruses

Virome datasets of RNA viruses were previously obtained from 291 deep-sea sediment samples collected by our laboratory (Zhang et al., 2024b,a) (National Omics Data Encyclopedia database, accession numbers OEP002537, OEP003296, and OEP00006529). Deep-sea sediment samples were collected during the 22nd, 26th, 30th, 32nd, 34th, 39th, 40th, 45th and 75th cruises of the Dayang No. 1 survey of China from 2010 to 2022. These samples were geographically distributed throughout the Pacific, Atlantic and Indian Oceans, encompassing seven distinct deep-sea ecosystems (seamount, ocean basin, cold seep, hydrothermal vent, hadal trench, polymetallic nodule area and mid ocean ridge; Supplementary Table S4). Viral particles were isolated and enriched from deep-sea sediment samples using a sequential concentration protocol. Briefly, 20 g of sediment was resuspended in 10 mL of 0.015 μm pre-filtered Milli-Q water with glass beads and agitated at 4 C for 30 min to elute viral particles. The suspension was centrifuged at 5,000 × g for 20 min at 4 C, and the supernatant was retained. The pellet was re-eluted six times under the same conditions, and all supernatants were pooled. The pooled supernatant was clarified by centrifugation at 5,000 × g for 10 min at 4 C, filtered through a 0.22 μm membrane, and then supplemented with PEG 6,000 to a final concentration of 10% (w/v) for overnight precipitation at 4 C. Finally, viral particles were pelleted by ultracentrifugation at 200,000 × g for 2 h at 4 C and resuspended in SM buffer. The resuspended viral particles were either used immediately for TEM observation or stored at −80 C until further processing. For metagenomic sequencing, viral RNA was extracted from the concentrated particles following DNase I treatment to remove extraneous DNA, followed by reverse transcription and isothermal amplification using the GenomiPhi^TM^ V2 DNA amplification kit (GE Healthcare Life Sciences, Buckinghamshire, UK) with random hexamers. The amplified products were then used for RNA viral metagenome sequencing as previously described (Zhang et al., 2024b,a).

### 4.2 Sequence quality assessment and quality control

To ensure the accuracy and reliability of downstream bioinformatic analyses, raw sequencing data were systematically processed to generate high-quality clean reads. The quality-control workflow included adapter removal, end trimming, overlap-based correction, ambiguous-base filtering, deep quality filtering and host-contaminant removal. Briefly, residual adapter sequences were first identified and removed to eliminate artificial sequence interference introduced during sequencing. Fastp v0.23.0 (https://github.com/OpenGene/fastp) was then used to uniformly trim the 5′ and 3′ ends of all reads, thereby removing sequencing cycles with unstable quality and minimizing the influence of quality deterioration at read ends. For paired-end reads, overlapping regions were examined, and inconsistent bases within these regions were corrected when applicable to improve base accuracy. Reads containing excessive ambiguous bases (N) were subsequently discarded.

Deep filtering was further performed using SOAPnuke v2.1.8 (https://github.com/BGI-flexlab/SOAPnuke) with the parameter “-q 0.2”, followed by secondary filtering based on Phred quality thresholds to remove residual sequencing errors. Finally, the resulting clean reads were mapped against the human reference genome using Bowtie2 v2.5.3 (http://bowtiebio.sourceforge.net/bowtie2/index.shtml), and reads with high mapping similarity were removed as host-derived or contaminant reads to ensure the species specificity and purity of the data used for downstream analyses.

### 4.3 Contig assembly and viral contig identification

Sequencing reads were quality-trimmed using Trimmomatic v0.33 with default parameters (Bolger et al., 2014), and the resulting high-quality reads were assembled into contigs using metaSPAdes v3.12.0 (Nurk et al., 2017). Contigs shorter than 300 bp were removed to ensure sequence quality and support downstream analyses, including gene prediction. The retained contigs were statistically evaluated and used for subsequent analyses.

Viral contigs were identified from the assembled contigs using commonly used viral detection tools, including VirSorter (Roux et al., 2015), VirFinder (Ren et al., 2017), VIBRANT (Kieft et al., 2020) and CAT (Von Meijenfeldt et al., 2019). These tools were used in a complementary manner to improve the accuracy of viral contig identification and reduce false positives. Considering that the hepatitis D virus genome, approximately 1.7 kb, represents one of the smallest known viral genomes (Ren et al., 2020), contigs with lengths ≥1.0 kb were selected for viral sequence screening.

Contigs ≥1.0 kb were screened according to the following criteria: (1) contigs classified as category 1 or 2 by VirSorter; (2) contigs with VirFinder scores ≥0.9 and *p* < 0.05; (3) contigs classified into VirSorter categories 1–6 and simultaneously showing VirFinder scores ≥0.7 with *p* < 0.05; and (4) all contigs identified by VIBRANT. Contigs that were not selected by VirSorter, VirFinder, or VIBRANT were further screened using CAT (Von Meijenfeldt et al., 2019). In the CAT-based screening, contigs with less than 40% of their genomic regions classified as bacterial, archaeal or eukaryotic were considered viral contigs.

Viral contigs identified by VirSorter, VirFinder, VIBRANT, and CAT were merged, and duplicate sequences were removed. Candidate viral sequences were further annotated and validated by comparison against the viral RefSeq database (https://www.ncbi.nlm.nih.gov/refseq/), the Kyoto Encyclopedia of Genes and Genomes database, the Pfam protein family database and the Viral Orthologous Groups database, enabling systematic identification of viral contigs.

### 4.4 Identification of viral operational taxonomic units

To systematically assess viral genome completeness and classify the identified viral genome sequences at the species level, all viral genome sequences were clustered using the ANI_cluster.py script in CheckV v0.8.1 (https://bitbucket.org/berkeleylab/checkv). Viral operational taxonomic units (vOTUs) were constructed at the species level based on a dual-threshold criterion of 95% average nucleotide identity (ANI) and 85% genome coverage.

### 4.5 Cell culture

HEK-293T cells were cultured in Dulbecco's Modified Eagle Medium (DMEM; Gibco, USA) supplemented with 10% fetal bovine serum (FBS) and 1% penicillin streptomycin solution (Gibco). Cells were maintained at 37 C in a humidified atmosphere with 5% CO_2_.

### 4.6 Prediction of ribozymes

Hammerhead ribozymes were predicted using the online RNABOB software (http://eddylab.org/software.html) with diverse descriptors for hammerhead ribozymes containing three helices and a catalytic core composed of conserved nucleotides. Because RNABOB does not perform score-based sequence alignment, no e-value, sequence identity and coverage thresholds were applied. Only sequences satisfying the predefined descriptor constraints, including stem, loop and conserved catalytic motif constraints, were retained for downstream analyses. Secondary structures of three types of hammerhead ribozymes were determined using RNABOB analysis. The descriptors for the hammerhead search were listed as follows.


$$\begin{array}{r} \text{Canonical form}5^{\text{′}}-\left[\text{h}1\right]-\text{s}1-\left[\text{h}2\right]-\text{s}2-\left[2^{\text{′}}\right]-\text{s}3-\left[\text{h}3\right]\\ -\text{s}4-\left[3^{\text{′}}\right]-\text{s}5-\left[1^{\text{′}}\right]-3^{\text{′}} \end{array}$$

Where stem h1/h1′ (the complementary stem of h1) is NNN•NNN, h2/h2′ (the complementary stem of h2 is GNN•NNC, h3/3′ (the complementary stem of h3) is AN•NT, loop s1 is CYGANGA, loops s2 and s4 are N[98]N, loop s3 is GAA and loop s5 is A/C/T. N is A/C/G/T and [98] means 0 to 98 nucleotides. CYGANGA is the conserved catalytic motif. Y means C or U.

HHR II secondary structure:


$$\begin{array}{r} \text{Canonical form}5^{\text{′}}-\left[\text{h}1\right]-\text{s}1-\left[\text{h}2\right]-\text{s}2-\left[2^{\text{′}}\right]-\text{s}3-\left[\text{h}3\right]\\ -\text{s}4-\left[3^{\text{′}}\right]-\text{s}5-\left[1^{\text{′}}\right]-3^{\text{′}} \end{array}$$

Where stem h1/h1′ (the complementary stem of h1) is NNC•GNN, h2/h2′ (the complementary stem of h2) is AN•NU, h3/3′ (the complementary stem of h3) is NNN•NNN, loop s1 is GAA, loops s2 and s4 are N[98]N, loop s3 is A/C/T and loop s5 is CYGANGA. N is A/C/G/T and [98] means 0 to 98 nucleotides. CYGANGA is the conserved catalytic motif. Y means C or U.

HHR III secondary structure:


$$\begin{array}{r} \text{Canonical form}5^{\text{′}}-\left[\text{h}1\right]-\text{s}1-\left[\text{h}2\right]-\text{s}2-\left[2^{\text{′}}\right]-\text{s}3-\left[\text{h}3\right]\\ -\text{s}4-\left[3^{\text{′}}\right]-\text{s}5-\left[1^{\text{′}}\right]-3^{\text{′}} \end{array}$$

Where stem h1/h1′ (the complementary stem of h1) is NNU•ANN, h2/h2′ (the complementary stem of h2) is NNN•NNN, h3/3′ (the complementary stem of h3) is GNN•NNC, loop s1 is A/C/T, loops s2 and s4 are N[98]N, loop s3 is CYGANGA and loop s5 is GAA. N is A/C/G/T and [98] means 0 to 98 nucleotides. CYGANGA is the conserved catalytic motif. Y means C or U.

### 4.7 Engineering of ribozymes

The binding arms of the ribozymes DP162 and DP163 were engineered to target a specific 10-nucleotide sequence5′-CCAGCTCACT3′ within the HIV (human immunodeficiency virus)-1 rev gene (the engineered DP163),5′-CATCACCACA3′ within the HSV (herpes simplex virus)-1 *ICP0* gene (the engineered DP162-1) and 5′-GAGCCTTCTG-3′ of the nectin-1 gene (the engineered DP162-2), respectively. The conserved regions and stem loop structure essential for the catalytic activity of the ribozymes were preserved in all engineered variants.

To generate catalytic core deletion mutant of ribozyme DP 162 or DP163, a DNA template (DP162, 5′-TAATACGACTCACTATAGGGAGTGCGATGAATTCGGATAT GACAAGGTCGCGGCGCGTATCGCATGTCTGACTTTGTATT-3′; DP163, 5′-TAATACGACTCACTATAGGGTCAATGGTTTTTGTTGTACAAACAAAC-CATACTTTATCC CAAACTGATACCAACTAGCGTGGCTTTTTGGCG-GAGCTAAATGGAGCGCAATACTTTAGCCGGCTTTTT TGTTGG-3′) was synthesized. Each DNA template contained the T7 promoter sequence (5′-TAATACGACTCACTATAG-3′) and the catalytic core free DP162 or DP163 sequence. The catalytic core region of DP162 or DP163 comprised conserved region I (5′-CUGAUGA-3′), conserved region II (5′-GAAA-3′) and conserved region III (5′-CGAA-3′). RNA transcripts were generated from the DNA templates using T7 RNA polymerase *in vitro* transcription to produce catalytic core deletion mutant of ribozyme DP 162 (DP162Δcat) or DP163 (DP163ΔCat).

To confirm the deep-sea ribozymes, a known ribozyme HH16 (Hertel et al., 1994) was engineered as a positive ribozyme. HH16 is a well-characterized minimal hammerhead ribozyme with a 38-nucleotide length, consisting of a 38-nucleotide ribozyme strand (E) annealed to a 17-nucleotide full length substrate. HH16 is routinely used as a reference control in hammerhead ribozyme experiments because it is one of the most extensively characterized minimal hammerhead ribozymes, with its cleavage kinetics, thermodynamic parameters, and substrate binding properties thoroughly documented under standard reaction conditions. Its well-defined canonical three helix junction structure and reproducible kinetic behavior make it an ideal benchmark for directly comparing the performance of novel ribozyme variants. The substrate strand of HH16 was engineered and synthesized to target *pol* gene (5′-AAUCUGAUGAGGCCGAAAGGCCGAAAAACUA-3′) or *ICP4* gene (5′-UGCCUGAUGAGGCCGAAAG GCCGAAAGCGGC-3′). This design allowed a direct comparison of catalytic performance between the positive ribozyme (HH16) benchmark and the deep-sea derived ribozymes.

### 4.8 HIV-1-based VSV-G pseudotyped lentivirus production and infection

For lentivirus production, the plasmid PLVX-shRNA encoding ZsGreen (a green fluorescent protein variant), the packaging plasmid psPAX2, which encodes HIV-1-derived *gag* and *pol* genes, and the envelope plasmid pMD2.G, which encodes the vesicular stomatitis virus G protein (VSV-G), (GenePharma Co., Ltd., Shanghai, China) were co-transfected into HEK-293T cells at a ratio of 4:3:1 using Lipofectamine 2,000 (Invitrogen, USA). At 48 h after co-transfection, the supernatant was filtered through a 0.45 μm filter to remove cellular debris, then concentrated using 100 kDa cutoff ultrafiltration. Concentrated viral particles were harvested for subsequent infection experiments.

### 4.9 Antiviral activity assay of ribozyme

HEK-293T cells were seeded at 1 × 10^5^ cells per well in a 24-well plate. The cells were transfected with a ribozyme or a random RNA (5′-CGACGGAATAATAGCATAGTGGGCGATAGAATATTTG CCCACGAAATCCTTGTTGTGACCCCTCATCTT TTATACCTTCTGTCGTGCTTAGTCCTATTGCGTGAG-TAGACGTACATAAGAGAAATAGTA3′) at a final concentration of 50 nM using Lipofectamine 2,000 (Invitrogen, USA) according to the manufacturer's instructions and cultured for 6 h, with reference to previously reported hammerhead ribozyme transfection conditions (Wang et al., 2024). Subsequently, the cells were infected with HIV-1 pseudovirus at a multiplicity of infection (MOI) of 10. At different time post-infection (24, 48 and 72 h), virus copies and cellular fluorescence were quantified.

### 4.10 Quantitative real-time PCR

Total RNAs were extracted from virus-infected cells using TRIzol extraction kit (TransGen Biotech, Beijing, China). The extracted RNAs were reversely transcribed into complementary DNA (cDNA) using a reverse transcription kit (Vazyme Biotech Corporation, Nanjing, China). Then the virus copies were quantified based on a standard curve using HIV-1 specific primers (5′-GGAGTTGTGGCCCGTTGTCAGG-3′ and 5′-CGGGAAGGAAGGTCCGCTGGAT-3′). The standard curve was constructed with the woodchuck hepatitis virus post transcriptional regulatory element of HIV-1. Quantitative real-time PCR was performed using SYBR Green PCR Master Mix (Vazyme Biotech Corporation). PCR conditions were initial denaturation at 95 C for 30 s, followed by 40 cycles of denaturation at 95 C for 10 s and annealing/extension at 60 C for 30 s. Melting curve analysis was performed at the end of each run to confirm amplification specificity.

To detect the mRNA level of a gene, quantitative real-time PCR was carried out using gene specific primers (GAPDH, 5′-GAGAAGGCTGGGGCTCATTT-3′ and 5′-GTGATGGCATGGACTGTGG-3′; pol, 5′-GGGGATGTGCTGCAAGGCGATT-3′ and 5′-CACTCATTAGGCACCCCAGGCT-3′; IFN-α, 5′-AACTCCCCTGATGAATG CGG−3′ and 5′-AGCAGGGGTGAGAGTCTTTG-3′; IFN-β, 5′-AGTAGGCGACACTGTTCGTG-3′ and 5′-GTCTCATTCCAGCCAGTGCT-3′; IFN-γ, 5′-GAGGTCGAAGAGCATCCCAG−3′ and 5′-GCTTAGGTTGGCTGCCTAGT−3′; DP163, 5′-AACT GATAC-CGAAAAACTAGCGTGG-3′ and 5′-GCCGGCTAAAGTATTTTCGGCGC-3′; CXCL10, 5′-GCTTCCAAGGATGGACCACA-3′ and 5′-GCAGGGTCGAACAT CCACT-3′; IL6, 5′-CCACCGGGAACGAAAGAGAA-3′ and 5′-GAGAAGGCAACTGGACCGAA-3′; TNF-α, 5′-CTCTTCTGCCTGCTGCACTTTG−3′ and5′-ATGGGCTACAGGCTT-GTCACTC-3′; ISG15, 5′-AGCGAACTCATCTTTGCCAGTA-3′ and 5′-CAGCTCTGACACCGACATGGA-3′; ISG54,5′-GAAGACAAGGCCATCCAC CA-3′ and 5′-CAGCTCCTGAAGGAATGCCA-3′; RIG-I,5′-CATGTCCACCTTCAGAAGTGTCT-3′ and 5′-CCCCTTTTGTCCTTGTGGGA-3′; ICP4, 5′-TATATGAGCCCGAGGACGCCCC-3′ and 5′-GGAAAAAGGACAGGGACGGCCG-3′; DP162, 5′-GACTCACTATAGGGAGTGCCTGA-3′ and 5′-GATACGCGCCGCTTTCGACCTT-3′). Glyceraldehyde 3 phosphate dehydrogenase (GAPDH) served as the house keeping gene.

### 4.11 Western blot

Proteins were separated by sodium dodecyl sulfate polyacrylamide gel electrophoresis (SDS PAGE) and then transferred to a nitrocellulose membrane (Cytiva Life Sciences, Shanghai, China). After blocking in 5% (w/v) nonfat milk for 2 h at room temperature, the membrane was incubated overnight with a primary antibody at 4 C. Subsequently the membrane was incubated with the horseradish peroxidase (HRP) conjugated secondary antibody for 2 h at room temperature. The signals on the membrane were examined using an enhanced chemiluminescence (ECL) detection system (Science and Technology Co. Ltd, Shanghai, China).

### 4.12 *In vitro* ribozyme cleavage assay

Ribozyme mediated cleavage reactions were performed in a reaction mixture consisting of 2 μM ribozyme, 2 μM target RNA substrate, 50 mM Tris HCl (pH 7.5) and 10 mM MgCl_2_. The mixture was heated to 85 C for 2 min, followed by gradual cooling to room temperature to allow proper RNA folding. The cleavage reaction was initiated by incubation at 37 C for 1 h and terminated by adding ethylenediaminetetraacetic acid (EDTA) at a final concentration of 50 mM. Samples were then heated to 95 C for 3 min to denature RNA secondary structures and immediately placed on ice. The product was analyzed by denaturing 15% polyacrylamide gel electrophoresis (PAGE) in the presence of 8 M urea. This protocol was adapted from previously established methods for *in vitro* characterization of hammerhead ribozyme activity (Hertel et al., 1994; Liu et al., 2017).

### 4.13 Statistical analysis

All experiments were performed with three independent biological replicates (*n* = 3). Numerical data were presented as mean ± standard deviation (SD). Statistical significance was determined using one way analysis of variance (ANOVA) and Student's t test for pairwise comparisons between treatments.

## Data Availability

The data that support the findings of this study are openly available in the National Omics Data Encyclopedia database (https://www.biosino.org/node/home) under accession numbers OEP002537, OEP003296, and OEP00006529.
