# Supplementary material for: Systematic discovery of novel ribozymes with gene silencing properties from global deep-sea RNA viromes
Source: Front Microbiol. 2026 Aug 4;17:1866359. doi: 10.3389/fmicb.2026.1866359 (PMC13481778; doi:10.3389/fmicb.2026.1866359)
Supplement: Supplementary file 1 [file Supplementary_file_1.docx]

**Supplementary figures**

**Fig S1. *In vitro* cleavage of pol mRNA by the ribozyme DP163.** The ribozyme DP163 was incubated with the *pol* mRNA. The mixture was examined using denaturing polyacrylamide gel electrophoresis. DP163 alone and the *pol* mRNA alone were used as controls.

**Fig S2. *In vitro* cleavage analysis of the ribozyme DP162 on its target mRNA.** The catalytic activity of DP162 was evaluated using the *in vitro* cleavage assays. The ribozyme DP162 was incubated with the ICP4 mRNA. The cleavage products were analyzed using the denaturing polyacrylamide gel electrophoresis. The purified DP162 RNA and ICP4 mRNA alone served as controls.

Fig S1

Fig S2
